# Hormone Receptor Status May Impact the Survival Benefit Between Medullary Breast Carcinoma and Atypical Medullary Carcinoma of the Breast: A Population-Based Study

**DOI:** 10.3389/fonc.2021.677207

**Published:** 2021-07-06

**Authors:** Wenxing Qin, Feng Qi, Mengzhou Guo, Liangzhe Wang, Yuan-Sheng Zang

**Affiliations:** ^1^ Department of Oncology, Second Affiliated Hospital of Naval Medical University, Shanghai, China; ^2^ Liver Cancer Institute, Zhongshan Hospital, Fudan University, Shanghai, China; ^3^ Department of Pathology, Second Affiliated Hospital of Naval Medical University, Shanghai, China

**Keywords:** hormone receptor status, breast cancer, medullary carcinoma, nomogram, atypical medullary carcinoma of the breast

## Abstract

**Background:**

A rare subtype of breast cancer, atypical medullary carcinoma of the breast (AMCB), shows a highly adverse prognosis compared to medullary carcinoma of the breast (MBC). The current study aimed to establish a correlated nomogram for the identification of the prognostic factors of AMCB and MBC.

**Methods:**

Kaplan–Meier and Cox regression analyses were applied to data acquired from the Surveillance, Epidemiology and End Results (SEER) database for 2004 to 2013 to analyse tumour characteristics and overall survival. Propensity score matching (PSM) analysis was performed to determine the overall survival (OS) among those with AMCB and MBC. A predictive nomogram was created, and the concordance index (C-index) was used to predict accuracy and discriminative ability.

**Results:**

A total of 2,001 patients from the SEER database were diagnosed with MBC between 2004 and 2013, including 147 patients diagnosed with AMCB. The number of diagnoses gradually increased in both groups. Cox analysis of multivariate and Kaplan–Meier analysis showed that older age (HR = 3.005, 95% CI 1.906–4.739) and later stage were significantly associated with poor prognosis, while cancer-directed surgery was an independent protective factor (HR = 0.252, 95% CI 0.086–0.740). In the HR-negative stratification analysis, older age (HR = 2.476, 95% CI 1.398–4.385), later stage and histological type (HR=0.381, 95% CI 0.198-0.734) were found to be independent prognostic factors for low standard survival. The log-rank analysis demonstrated significantly worse prognostic factors for patients with AMCB than for those with MBC (*P* = 0.004). A nomogram (C-index for survival = 0.75; 95% CI 0.69–0.81) was established from four independent prognostic factors after complete identification.

**Conclusions:**

MBC is rare, and cancer-directed surgery, older age, and later stage are independently linked with prognosis. In the HR negative population, AMCB patients show a worse survival gain than those with MBC.

## Introduction

Breast cancer is a major malignant tumour in women, ranking second in incidence among female malignant tumours. Medullary carcinoma of the breast (MBC) has been declared a special type of infiltrating breast cancer, accounting for approximately 5–7% of cases (1). The boundary of the typical medullary carcinoma of the breast (TMCB) is clearer, and a large number of lymphocytes infiltrate the interstitial substance; thus, it has a slower growth and more favourable prognosis (2). However, atypical medullary carcinoma of the breast (AMCB) is characterized by no obvious histologic boundaries and a poor prognosis.

Therefore, it is important to determine and characterize the prognostic factors for AMCB patients to facilitate diagnosis and clinical treatment. Although the cellular morphology of AMCB is essentially consistent with that of MBC, it fails to conform to the diagnostic criteria of MBC and features infiltrating tumour borders (3). Several studies have reported that the overall survival (OS) and disease-free survival (DFS) of MBC patients were closely related to age, race, local metastasis, distant metastasis, tumour size, hormone receptor status, and lymph node metastasis (4–7). In addition, HR status also affected the survival of MBC patients. Some studies found that MBC patients had a higher percentage of triple-negative status, and MBC patients with PR negativity exhibited good prognosis (8, 9). However, the prognostic factors of AMCB were not mentioned. To address this, we examined patients with AMCB and MBC of the breast cancer referring to the Surveillance, Epidemiology and End Results (SEER) database and offer a related retrospective assessment. We compared the overall survival, prognostic factors, and clinical features between MBC and AMCB patients. In addition, hormone receptor status was used as a stratification analysis to analyse the survival benefit of AMCB and MBC patients.

## Materials and Methods

### Patient Data

Data from patients diagnosed with breast cancer from 2004 to 2013 were downloaded using Stat version 8.2.1 of SEER. Patient data were included in this analysis if the following criteria were met: ages 18 to 79; breast cancer as the primary malignant cancer diagnosis; estrogen receptor (ER), progesterone receptor (PR) and human epidermal growth factor receptor 2 (Her2) status available; medullary carcinoma (based on ICD-O-3 8512/3) and pathological types including atypical medullary carcinoma (based on ICD-O-3 8513/3) if not specified (MBC-NOS, ICD-O-3 8510/3); and definite AJCC TNM stages; and histological grades I to IV. We excluded patients who did not have a clear tumour stage or a record of months of survival. Additionally, we also included data from patients exhibiting a history of breast cancer diagnosis prior to 2013 to ensure a sufficiently extensive follow-up period. The study was in compliance with the ethics statement of Changzheng Hospital.

### Statistical Analysis

The expression status of ER and PR was combined as the hormone receptor (HR) state. The expression of HR− was further defined as ER− and PR−, while the expression of HR+ was defined as ER+ and PR+. Unclear expression of ER or PR was defined as unknown. According to the pathological type, breast cancer patients were allocated into two groups: the AMCB and MBC groups. The t-test and, where necessary, the Mann–Whitney U test were employed to compare the homogeneity of variance and continuous variables in the normal distribution. Tukey’s test and one-way ANOVA were employed to compare multiple groups. The chi-square test was used to compare the clinical and demographic characteristics of the three groups. The Kaplan–Meier method was employed to plot the survival curve, and the log-rank test was used to adjust the unadjusted overall survival rates of different histological subtypes. Overall survival was defined as the period from diagnosis to the final follow-up or the complete disappearance of the tumour. The prognostic factors were computed using the Cox proportional hazards model, where HR was the 95% confidence interval. PSM analyses were performed based on age, race, sex, grade, laterality, AJCC stage, T category, N category, local treatment of the primary tumour, ER, PR and Her2 at a 1:1 ratio to adjust for the differences among the AMCB and MBC groups. All statistical analyses were computed using SPSS version 22.0 (IBM SPSS Statistics, Chicago, IL, US), and *P <*0.05 was considered statistically significant.

## Results

### Demographic Analysis of Patients With AMCB and MBC

**Figure 1:**
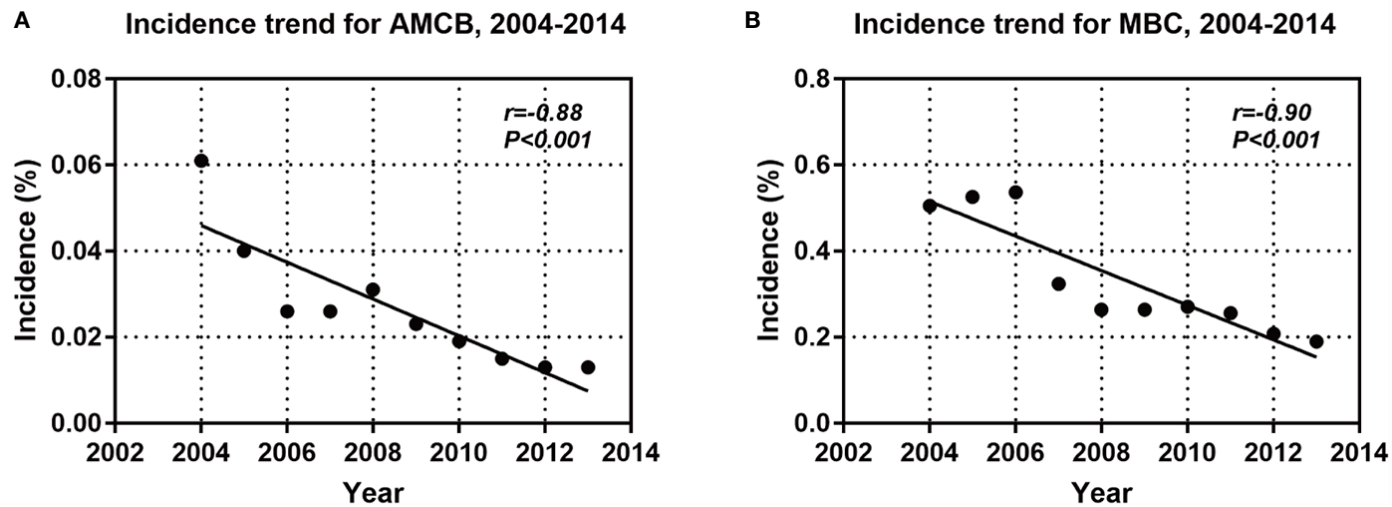
Incidence trends for AMCB **(A)** and MBC **(B)**.

A total of 2,001 eligible patients, including 1,863 MBC patients and 147 AMCB patients, were included in our study based on the inclusion criteria. The clinical and demographic characteristics of all patients in the SEER database with Histologic Type of Breast Cancer from 2004–2013 are summarized in **Table 1**. The X-tile analysis revealed that the best possible cut-off value to assess prognostic factors for age was 71 years. The comparison of MBC and AMCB showed no substantial differences in race, sex, age, degree of differentiation, tumour size, AJCC stage, lymph node metastasis, Her2 status, HR status, or surgical method. Similar to MBC, the rate of incidence of AMCB was high in triple-negative breast cancer. In molecular subtypes, three categories were defined (**Table 1**). Regarding Her2 status, the frequency of Her2-negative cases was higher in the MBC group than in the AMCB group (24.6% *vs* 17.7%, *P* = 0.108). Overall, the number of Her2-negative patients was higher than that of Her2-positive patients (24.1% *vs* 3.1%). However, 72.8% of cases were characterized as unknown, with neither Her2 negativity nor Her2 positivity. Due to limited data, the comparative analysis of the treatment method did not generate statistically significant results. Between 2004 and 2013, the overall incidence rate trends of AMCB and MBC decreased (AMBC: r = −0.88 and MBC: r = −0.90, *P <*0.001) (**Figure 1**).

**Table 1:** Patient characteristics with histologic type of breast cancer in the SEER database, 2004–2013.

Characteristics	Histologic type			
	AMCB	MBC	Total	*P*-value
**Number**	147	1,863	2,010	
**Age (years)**	53.26 ± 12.25	53.68 ± 13.00		0.707
**Marital status**				
Married	90 (61.2%)	1,018 (54.6%)	1,180 (55.1%)	0.298
Not married^a^	52 (35.4%)	777 (41.7%)	829 (41.2%)	
Unknown	5 (3.4%)	68 (3.7%)	73 (3.6%)	
**Race**				
White	100 (68.0%)	1,255 (67.4%)	1,355 (67.4%)	0.548
Black	33 (22.4%)	475 (25.5%)	508 (25.3%)	
Other^b^	14 (9.5%)	129 (6.9%)	143 (7.1%)	
Unknown	0 (0%)	4 (0.2%)	4 (0.2%)	
**Sex**				
Female	147 (100%)	1,858 (99.7%)	2,005 (99.8%)	0.529
Male	0 (0%)	5 (0.3%)	5 (0.2%)	
**Grade**				
Well differentiated	1 (0.7%)	13 (0.7%)	14 (0.7%)	0.715
Moderate	8 (5.4%)	102 (5.5%)	110 (5.5%)	
Poor	122 (83.0%)	1,481 (79.5%)	1,603 (79.8%)	
Unknown	16 (10.9%)	267 (14.3%)	283 (14.1%)	
**Laterality**				
Right	66 (44.9%)	926 (49.7%)	992 (49.4%)	0.485
Left	81 (55.1%)	935 (50.2%)	1,016 (50.5%)	
Bilateral	0 (0%)	2 (0.1%)	2 (0.1%)	
**AJCC stage**				
I	52 (35.4%)	699 (37.5%)	751 (37.4%)	0.293
II	80 (54.4%)	1,027 (55.1%)	1,107 (55.1%)	
III	12 (8.2%)	124 (6.7%)	136 (6.8%)	
IV	3 (2.0%)	13 (0.7%)	16 (0.8%)	
**T category**				
T0	0 (0%)	2 (0.1%)	2 (0.1%)	0.780
T1	65 (44.2%)	848 (45.5%)	913 (45.4%)	
T2	70 (47.6%)	909 (48.8%)	979 (48.7%)	
T3	10 (6.8%)	84 (4.5%)	94 (4.7%)	
T4	2 (1.4%)	16 (0.9%)	18 (0.9%)	
Tx	0 (0%)	4 (0.2%)	4 (0.2%)	
**N category**				
N0	111 (75.5%)	1,406 (75.5%)	1,517 (75.5%)	0.544
N1	25 (17.0%)	368 (19.8%)	393 (19.6%)	
N2	8 (5.4%)	63 (3.4%)	71 (3.5%)	
N3	3 (2.0%)	23 (1.2%)	26 (1.3%)	
Nx	0 (0%)	3 (0.2%)	3 (0.1%)	
**Local treatment of the primary tumour**				
Surgery	145 (98.6%)	1,838 (98.7%)	1,983 (98.7%)	0.985
No surgery	2 (1.4%)	25 (1.3%)	27 (1.3%)	
**ER**				
Positive	34 (23.1%)	388 (20.8%)	422 (21.0%)	0.752
Negative	105 (71.4%)	1,356 (72.8%)	1,461 (72.7%)	
Unknown	8 (5.4%)	119 (6.4%)	127 (6.3%)	
**PR**				
Positive	20 (13.6%)	246 (13.2%)	266 (13.2%)	0.812
Negative	119 (81.0%)	1,490 (80.0%)	1,609 (80.0%)	
Unknown	8 (5.4%)	127 (6.8%)	135 (6.7%)	
**Her2**				
Positive	3 (2.0%)	59 (3.2%)	62 (3.1%)	0.108
Negative	26 (17.7%)	458 (24.6%)	484 (24.1%)	
Unknown	118 (80.3%)	1,346 (72.2%)	1,464 (72.8%)	

a Including divorced, separated, single (never married), unmarried or domestic partner and widowed.

b >Including American Indian/Alaskan Native and Asian/Pacific Islander.

AJCC, American Joint Committee on Cancer; AMCB, atypical medullary carcinoma; MBC, medullary breast carcinoma; ER, oestrogen receptor; PR, progesterone receptor; Her2, human epidermal growth factor receptor 2.

### Survival and Prognostic Factors of AMCB and MBC Patients

**Figure 2:**
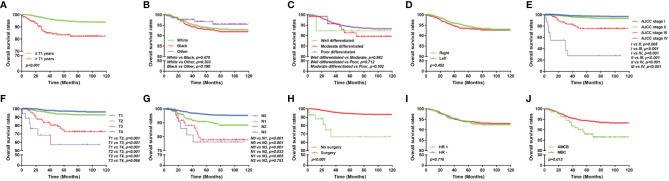
Overall survival of MBC and AMCB patients using the Kaplan–Meier estimator stratified by **(A)** age at diagnosis; **(B)** race; **(C)** grade; **(D)** laterality; **(E)** AJCC stage; **(F)** primary tumour size; **(G)** lymph node status; **(H)** surgery for primary tumour; **(I)** HR status; and **(J)** histological type.

The overall survival of MBC and AMCB patients was estimated using the Kaplan–Meier estimator. The survival curve was stratified by race, sex, age at diagnosis, laterality, AJCC stage, primary tumour size, primary tumour differential grade, surgery for the primary tumour, lymph node status, HR status, and histological type (**Figure 2**). As illustrated, older-aged patients had poorer survival (5-year overall survival rate: 83.3% vs 94.9%). The outcomes were extremely poor for patients with advanced cancer stages; the prognostic factors were the worst (27.5% 5-year overall survival rate) for MBC and AMCB patients with stage IV cancer, while the 5-year overall survival rates for stage I, II, and III cancers were 75.3, 94.5, and 97.3%, respectively (*P <*0.01, **Figures 2A, E**). Similarly, the overall survival rate of MBC and AMCB patients with smaller tumour sizes was significantly higher than that of patients with larger tumour sizes (*P <*0.01). The prognostic factors for MBC and AMCB patients were much worse when breast cancer cells were detected in lymph nodes (*P <*0.01) (**Figures 2F, G**). **Figure 2H** shows that the median overall survival for the MBC and AMCB patients significantly improved after cancer-directed surgery (*P <*0.01, mOS: 16.5 m *vs* 28.0 m). In the histological type analysis for OS, patients with MBC had better survival than those with AMCB (*P* = 0.013). The 5-year overall survival rates of MBC and AMCB patients were 94.3 and 87.8%, respectively.

The Cox regression models in both forms, i.e., univariate and multivariate analyses, were applied to the overall survival results to further analyse the prognostic factors. According to univariate factor analysis, as shown in **Table 2**, older age, larger tumour size, later stage, lymph node metastasis, and histological type of AMCB were significantly related to worse prognosis (*P <*0.01). Cancer-directed surgery was found to be significantly related to extended overall survival (*P <*0.01). After adjusting the multivariate analysis, as shown in **Table 2**, the independent prognostic factors of poorer survival in MBC and AMCB patients were older age and later stage only. However, cancer-directed surgery was found to be an independent protective factor that reduced the probability of death by 74.8% (HR = 0.252, 95% CI 0.086–0.740) in MBC and AMCB patients.

**Table 2:** Univariate and multivariate Cox proportional hazard analyses of the association of clinical characteristics with overall survival rates in patients with AMCB and MBC.

Variance	Univariate HR (95% CI)	*P* value	Multivariate HR (95% CI)	*P* value
**Age**				
≤71 years	1		1	
>71 years	3.603 (2.360–5.500)	<0.001	3.005 (1.906–4.739)	<0.001
**Race**				
White	1		1	
Black	1.163 (0.772–1.753)	0.470	1.076 (0.704–1.645)	0.734
Other	0.625 (0.253–1.546)	0.309	0.707 (0.283–1.768)	0.458
**Grade**				
Well differentiated	1		1	
Moderate	1.009 (0.126–8.068)	0.993	0.970 (0.110–8.546)	0.978
Poor	0.691 (0.096–4.965)	0.713	0.597 (0.076–4.713)	0.625
**Laterality**				
Right			1	
Left	1.154 (0.794–1.676)	0.453	0.987 (0.666–1.461)	0.947
Bilateral	17.641 (2.423–128.446)	0.005	0.258 (0.005–14.380)	0.509
**AJCC stage**				
I	1		1	
II	2.066 (1.229–3.472)	0.006	0.674 (0.255–1.783)	0.426
III	9.083 (5.025–16.416)	<0.001	0.813 (0.187–3.541)	0.783
IV	53.150 (24.633–114.681)	<0.001	13.065 (3.639–46.904)	<0.001
**Stage T**				
T0	1		1	
T1	0.027 (0.006–0.114)	<0.001	0.132 (0.015–1.174)	0.069
T2	0.058 (0.014–0.237)	<0.001	0.340 (0.042–2.751)	0.312
T3	0.232 (0.054–0.996)	0.049	0.839(0.094–7.454)	0.875
T4	0.542 (0.109–2.691)	0.454	1.511 (0.144–15.875)	0.731
**Stage N**				
N0	1		1	
N1	2.467 (1.626–3.745)	<0.001	1.951 (1.155–3.294)	0.012
N2	4.924 (2.642–9.177)	<0.001	2.918 (0.927–9.184)	0.067
N3	5.851 (2.345–14.600)	<0.001	3.459 (0.968–12.355)	0.056
**Local treatment of the primary tumour**				
None	1		1	
Surgery	0.135 (0.059–0.307)	<0.001	0.252 (0.086–0.740)	0.012
**HR**				
Positive	1		1	
Negative	0.928 (0.620–1.388)	0.716	1.055 (0.685–1.626)	0.808
**Histologic type**				
AMCB	1		1	
MBC	0.508 (0.295–0.874)	0.015	0.684 (0.371–1.260)	0.223

AMCB, atypical medullary carcinoma; MBC, medullary breast carcinoma; HR, hormone receptor.

PSM analysis was performed to adjust for the unmatching cohort, and a total of 147 AMCB patients were matched with 147 MBC patients (1:1) (**Table 3**). According to univariate and multivariate analyses, as shown in **Table 4**, compared with MBC, histological type of AMCB was identified as an independent risk factor (HR = 4.767, 95% CI 3.408–6.669).

**Table 3:** Patient characteristics with histologic type of breast cancer after propensity score matching in the SEER database, 2004–2013.

Characteristics	Histologic type			
	AMCB	MBC	Total	*P* value
**Number**	147	147	294	
**Age (years)**	53.26 ± 12.25	62.10± 14.46		<0.001
**Marital status**				
Married	90 (61.2%)	31 (21.1%)	121 (41.2%)	<0.001
Not married^a^	52 (35.4%)	92 (62.6%)	144 (49.0%)	
Unknown	5 (3.4%)	24 (16.3%)	29 (9.9%)	
**Race**				
White	100 (68.0%)	106 (72.1%)	206 (70.1%)	0.180
Black	33 (22.4%)	35 (23.8%)	68 (23.1%)	
Other^b^	14 (9.5%)	6 (4.1%)	20 (6.8%)	
**Sex**				
Female	147 (100%)	142 (96.6%)	289 (98.3%)	0.024
Male	0 (0%)	5 (3.4%)	5 (1.7%)	
**Grade**				
Well differentiated	1 (0.7%)	0 (0.7%)	1 (0.3%)	0.194
Moderate	8 (5.4%)	5 (3.4%)	13 (4.4%)	
Poor	122 (83.0%)	115 (78.2%)	237 (80.6%)	
Unknown	16 (10.9%)	27 (18.4%)	43 (14.6%)	
**Laterality**				
Right	66 (44.9%)	99 (67.3%)	165 (56.1%)	<0.001
Left	81 (55.1%)	47 (32.0%)	128 (43.5%)	
Bilateral	0 (0%)	1 (0.7%)	1 (0.3%)	
**AJCC stage**				
I	52 (35.4%)	82 (55.8%)	134 (45.6%)	0.003
II	80 (54.4%)	59 (40.1%)	139 (47.3%)	
III	12 (8.2%)	5 (3.4%)	17 (5.8%)	
IV	3 (2.0%)	1 (0.7%)	4 (1.4%)	
**T category**				
T1	65 (44.2%)	92 (62.6%)	157 (53.4%)	0.009
T2	70 (47.6%)	46 (31.3%)	116 (39.5%)	
T3	10 (6.8%)	9 (6.1%)	19 (6.5%)	
T4	2 (1.4%)	0 (0.0%)	2 (0.7%)	
**N category**				
N0	111 (75.5%)	115 (78.2%)	226 (76.9%)	0.293
N1	25 (17.0%)	27 (18.4%)	52 (17.7%)	
N2	8 (5.4%)	2 (1.4%)	10 (3.4%)	
N3	3 (2.0%)	2 (1.4%)	5 (1.7%)	
Nx	0 (0%)	1 (0.7%)	1 (0.3%)	
**Local treatment of the primary tumour**				
Surgery	145 (98.6%)	143 (97.3%)	288 (98.0%)	0.409
No surgery	2 (1.4%)	4 (2.7%)	6 (2.0%)	
**ER**				
Positive	34 (23.1%)	19 (12.9%)	53 (18.0%)	0.068
Negative	105 (71.4%)	117 (79.6%)	222 (75.5%)	
Unknown	8 (5.4%)	11 (7.5%)	19 (6.5%)	
**PR**				
Positive	20 (13.6%)	21 (14.3%)	41 (13.9%)	0.867
Negative	119 (81.0%)	116 (78.9%)	235 (79.9%)	
Unknown	8 (5.4%)	10 (6.8%)	18 (6.1%)	
**Her2**				
Positive	3 (2.0%)	50 (34.0%)	53 (18.0%)	<0.001
Negative	26 (17.7%)	75 (51.0%)	101 (34.4%)	
Unknown	118 (80.3%)	22 (15.0%)	140 (47.6%)	

a Including divorced, separated, single (never married), unmarried or domestic partner and widowed.

b Including American Indian/Alaskan Native and Asian/Pacific Islander.

AJCC, American Joint Committee on Cancer; AMCB, atypical medullary carcinoma; MBC, medullary breast carcinoma; ER, oestrogen receptor; PR, progesterone receptor; HER2, human epidermal growth factor receptor 2.

**Table 4:** Univariate and multivariate Cox proportional hazard analyses of the association of clinical characteristics with overall survival rates in patients with AMCB and MBC after propensity score matching.

Variance	Univariate HR (95% CI)	*P* value	Multivariate HR (95% CI)	*P* value
**Age**				
≤71 years	1		1	
>71 years	1.169 (0.872–1.568)	0.296	0.549 (0.389–0.774)	<0.001
**Race**				
White	1		1	
Black	0.825 (0.615–1.107)	0.199	0.856 (0.619–1.184)	0.349
Other	0.751 (0.456–1.237)	0.261	1.006 (0.576–1.757)	0.984
**Grade**				
Well differentiated	1		1	
Moderate	1.763 (0.225–13.805)	0.589	1.625 (0.199–13.285)	0.650
Poor	2.275 (0.318–16.264)	0.413	1.678 (0.213–13.209)	0.623
**Laterality**				
Right			1	
Left	0.869 (0.681–1.109)	0.260	1.159 (0.889–1.511)	0.275
Bilateral	3.420 (0.474–24.684)	0.223	3.910 (0.379–40.316)	0.252
**AJCC stage**				
I	1		1	
II	0.893 (0.696–1.145)	0.372	1.535 (0.808–2.916)	0.191
III	0.886 (0.500–1.569)	0.678	1.978 (0.320–12.220)	0.463
**Stage T**				
T1	1		1	
T2	0.907 (0.706–1.165)	0.445	0.794 (0.449–1.405)	0.428
T3	0.902 (0.488–1.668)	0.743	0.730 (0.298–1.788)	0.491
T4	0.727 (0.102–5.207)	0.751	1.032 (0.123–8.691)	0.977
**Stage N**				
N0	1		1	
N1	1.013 (0.740–1.386)	0.938	0.826 (0.527–1.295)	0.405
N2	0.932 (0.459–1.893)	0.846	0.851 (0.140–5.182)	0.861
N3	0.659 (0.211–2.063)	0.474	0.604 (0.080–4.537)	0.624
**Local treatment of the primary tumour**				
Surgery	1		1	
None	1.197 (0.445–3.218)	0.721	1.105 (0.338–3.609)	0.868
**HR**				
Positive	1		1	
Negative	1.142 (0.874–1.492)	0.329	1.025 (0.765–1.372)	0.870
**Histologic type**				
MBC	1		1	
AMCB	3.025 (2.340–3.911)	<0.001	4.767 (3.408–6.669)	<0.001

AMCB, atypical medullary carcinoma; MBC, medullary breast carcinoma; HR, hormone receptor.

### Baseline Characteristics and Survival Benefits in the Hormone Receptor Subgroups

**Figure 3:**
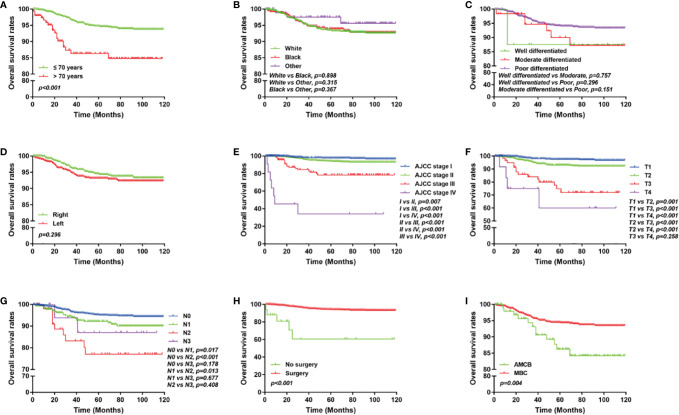
Overall survival of MCB and AMCB patients using the Kaplan–Meier estimator with HR negativity stratified by **(A)** age at diagnosis; **(B)** race; **(C)** grade; **(D)** laterality; **(E)** AJCC stage; **(F)** primary tumour size; **(G)** lymph node status; **(H)** surgery for primary tumour; **(I)** and histological type.

We analyzed the characteristics of the patients belonging to the HR-negative subgroup, which included 1,291 MBC and 102 AMCB patients (**Table 5**). The X-tile analysis revealed that the best possible cut-off value to assess prognostic factors for age was 71 years. The results were consistent with the entire population, and the comparison of MBC and AMCB showed no significant differences in sex, race, age, AJCC stage, degree of differentiation, tumour size, lymph node metastasis, or surgical method. The results of the Kaplan–Meier estimator and the Cox regression including univariate and multivariate analyses showed that later stage, older age, and histological type of AMCB were independently related to shortened OS, while surgery was independently related to prolonged overall survival (*P <*0.01, **Figure 3** and **Table 6**). The MBC group showed a better survival benefit than the AMCB group, with a lower hazard ratio of 0.38 (95% CI, 0.198–0.734, *P* = 0.004).

**Table 5:** HR (−) patient characteristics with AMCB and MBC.

Characteristics	Histologic type			
	AMCB	MBC	Total	*P*-value
**Number**	102	1,291	1,393	
**Age (years)**	53.31 ± 11.76	53.60 ± 12.93		0.830
**Marital status**				
Married	65 (63.7%)	716 (55.5%)	781 (56.1%)	0.269
Not married^a^	34 (33.3%)	527 (40.8%)	561 (40.3%)	
Unknown	3 (2.9%)	48 (3.7%)	51 (3.7%)	
**Race**				
White	73 (71.6%)	856 (66.3%)	929 (66.7%)	0.357
Black	20 (19.6%)	350 (27.1%)	370 (26.6%)	
Other^b^	9 (8.8%)	84 (6.5%)	93 (6.7%)	
Unknown	0 (0%)	1 (0.1%)	1 (0.1%)	
**Sex**				
Female	102 (100%)	1,290 (99.9%)	1,392 (99.9%)	0.779
Male	0 (0%)	1 (0.1%)	1 (0.1%)	
**Grade**				
Well differentiated	0 (0.0%)	9 (0.7%)	9 (0.6%)	0.715
Moderate	6 (5.9%)	54 (4.2%)	60 (4.3%)	
Poor	82 (80.4%)	1,052 (81.5%)	1,134 (81.4%)	
Unknown	14 (13.7%)	176 (13.6%)	190 (13.6%)	
**Laterality**				
Right	49 (48.0%)	645 (50.0%)	694 (49.8%)	0.894
Left	53 (52.0%)	645 (50.0%)	698 (50.1%)	
Bilateral	0 (0%)	1 (0.1%)	1 (0.1%)	
**AJCC stage**				
I	35 (34.3%)	479 (37.1%)	514 (36.9%)	0.490
I	59 (57.8%)	712 (55.2%)	771 (55.3%)	
III	6 (5.9%)	91 (7.0%)	97 (7.0%)	
IV	2 (2.0%)	9 (0.7%)	11 (0.8%)	
**T category**				
T0	0 (0%)	1 (0.1%)	1 (0.1%)	0.727
T1	45 (44.1%)	596 (46.2%)	641 (46.0%)	
T2	50 (49.0%)	626 (48.5%)	676 (48.5%)	
T3	7 (6.9%)	54 (4.2%)	61 (4.4%)	
T4	0 (0%)	12 (0.9%)	12 (0.9%)	
Tx	0 (0%)	2 (0.2%)	2 (0.1%)	
**N category**				
N0	81 (79.4%)	967 (74.9%)	1,048 (75.2%)	0.569
N1	15 (14.7%)	259 (20.1%)	274 (19.7%)	
N2	4 (3.9%)	49 (3.8%)	53 (3.8%)	
N3	2 (2.0%)	16 (1.2%)	18 (1.3%)	
**Local treatment of the primary tumour**				
No surgery	0 (0%)	17 (1.3%)	17 (1.2%)	0.487
Surgery	102 (100%)	1,273 (98.6%)	1,375 (98.7%)	
Unknown	0 (0%)	1 (0.1%)	1 (0.1%)	

a Including divorced, separated, single (never married), unmarried or domestic partner and widowed.

b Including American Indian/Alaskan Native and Asian/Pacific Islander.

AJCC, American Joint Committee on Cancer; AMCB, atypical medullary carcinoma; MBC, medullary breast carcinoma.

**Table 6:** Univariate and multivariate Cox proportional hazard analyses of the association of clinical characteristics with overall survival rates in HR (−) patients with AMCB and MBC.

Variance	Univariate HR (95% CI)	*P* value	Multivariate HR (95% CI)	*P* value
**Age**				
≤70 years	1		1	
>70 years	3.087 (1.822–5.232)	<0.001	2.476 (1.398–4.385)	0.002
**Race**				
White	1		1	
Black	0.968 (0.585–1.602)	0.899	0.981 (0.585–1.645)	0.943
Other	0.556 (0.174–1.779)	0.323	0.588 (0.179–1.925)	0.380
**Grade**				
Well differentiated	1		1	
Moderate	0.675 (0.081–5.608)	0.716	3.482 (0.337–36.016)	0.295
Poor	0.368 (0.051–2.655)	0.321	1.486 (0.161–13.749)	0.727
**Laterality**				
Right			1	
Left	1.268 (0.812–1.981)	0.297	1.134 (0.705–1.822)	0.605
**AJCC stage**				
I	1		1	
II	2.368 (1.247–4.498)	0.008	0.477 (0.131–1.743)	0.263
III	8.844 (4.222–18.524)	<0.001	0.376 (0.056–2.542)	0.316
IV	56.164 (22.067–142.943)	<0.001	13.166 (2.922–59.331)	0.001
**Stage T**				
T0	1		1	
T1	0.029 (0.004–0.219)	0.001	0.043 (0.004–0.468)	0.010
T2	0.076 (0.010–0.555)	0.011	0.178 (0.020–1.606)	0.124
T3	0.298 (0.039–2.272)	0.243	0.747 (0.076–7.356)	0.802
T4	0.567 (0.063–5.079)	0.612	1.016 (0.080–12.941)	0.990
**Stage N**				
N0	1		1	
N1	1.842 (1.106–3.067)	0.019	1.472 (0.751–2.882)	0.260
N2	4.780 (2.336–9.781)	<0.001	4.377 (1.116–17.166)	0.034
N3	2.541 (0.617–10.477)	0.197	1.992 (0.329–12.077)	0.454
**Local treatment of the primary tumour**				
None	1		1	
Surgery	0.099 (0.040–0.246)	<0.001	0.066 (0.022–0.195)	<0.001
**Histologic type**				
AMCB	1		1	
MBC	0.416 (0.225–0.770)	0.005	0.381 (0.198–0.734)	0.004

AMCB, atypical medullary carcinoma; MBC, medullary breast carcinoma.

### Prognostic Nomogram for AMCB and MBC Patients With Hormone Receptor Negativity

**Figure 4:**
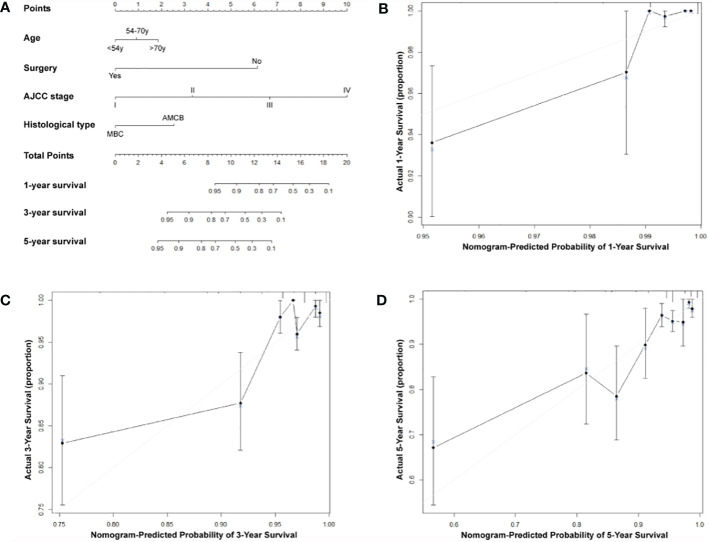
Predictive nomogram assessed by clinical characteristics for 1-, 3-, and 5-year overall survival in MBC and AMCB patients who were HR-negative. **(A)** The probability of 1-, 3-, or 5-year overall survival was estimated by connecting the probability scale to the total point scale through a vertical line. The calibration curves for overall survival at 1 year **(B)**, 3 years **(C)**, and 5 years **(D)** are shown.

A nomogram based on the prognostic factors that combined all significant independent variables for overall survival in the MBC and AMCB groups with HR negativity is shown in **Figure 4**. All patients were divided into three groups based on their age using the X-tile program, and the probabilities of 1-, 3-, or 5-year overall survival were determined. The optimal cut-points were 54 and 70 years. To determine the survival of MBC and AMCB patients more accurately with HR negativity, a nomogram based on the prognostic factors that included all significant independent variables in a multivariate Cox analysis was created (**Table 4**). Univariate and multivariate Cox analyses were used to calculate the association of the overall survival rate with the clinical characteristics of HR− patients with MBC and AMCB. The C-index for overall survival was found to be 0.75 (95% CI 0.69–0.81). As shown in **Figures 4B–D**, the actual observation and the prediction by the nomogram displayed optimal agreement based on the calibration plot for the probability of overall survival at 1, 3, or 5 years.

## Discussion

Medullary carcinoma is a distinct subgroup of breast cancers that makes up less than 5% of all advanced breast cancers. It has been considered that medullary breast carcinoma has a better prognosis than other common subtypes of histological breast cancer (10). In 1977, Ridolfi clarified the definition of MBC and proposed clear diagnostic criteria (six basic criteria) for the identification and diagnosis of medullary carcinoma (11). AMCB was defined to satisfy the first criterion of Ridolfi but not the remaining criteria. Through these precise diagnostic criteria, some institutions have reclassified breast medullary carcinoma (12) according to histopathological measures. Hormone receptors such as ER and PR are predictive prognostic factors and can serve as the foundations of a patient’s treatment for breast cancer. In previous studies, MBC was observed to have the lowest frequencies of ER, PR, and Her/neu-2 expression (13). ER positivity is infrequent in MBC, which is why previous studies reported only 547 patients (16.3%) with ER+ tumours. A positive ER in MBC patients is accompanied by the worst overall survival (5). Pinto et al. found no survival difference in patients with ER+/Her2/neu+ carcinomas and ER−/Her2/neu− carcinomas, indicating resistance to hormone therapy (14). Identification and characterization of prognostic factors for MBC patients will help in dictating diagnosis and suggesting more applicable treatments. In the current study, we tried to assess the clinicopathological features and prognostic factors of MBC and AMCB using data from patients in the SEER database from 2004 to 2013. MBC has many prognostic variables in common with ductal carcinoma infiltration. They both involve increasing age, the growing size of the tumour, metastasis at the lymph node, and the existence of cancer cells in distant lymph nodes, all of which shorten overall survival. Previous studies have identified numerous distinctive factors that are exclusively prognostic of survival in MBC (7, 15). Several factors, such as age, marital status, tumour size, stage, lymph node status, subtype of breast cancer, and radiation therapy, were substantially linked to overall survival in MBC (5). Our findings suggest that AMCB has a worse prognosis than MBC. Importantly, according to multivariate regression analysis, the prognosis of AMCB has close associations with age, stage, tumour size, surgical type, and hormone receptor positivity. In our research, the ratio of high-grade (grade III/IV) AMCB was 83.0%. Therefore, AMCB was considered a more aggressive tumour and was predominantly determined to be a high-grade tumour, similar to other studies (16). Moreover, the prevalence rate of HR-negative AMCB was 81.0%, which was more prevalent than HR-positive AMCB (13.6%), similar to other studies (17). According to further stratification studies, patients with hormone receptor-positive AMCB and MBC showed similar OS. In hormone receptor-negative patients, the prognosis of AMCB was significantly worse than that of MBC (data not shown). It was shown that the hormone receptor can be a clear independent prognostic factor for AMCB. It is important to realize that the clinicopathological features and prognostic factors of AMCB can serve as a reference for patients to provide more accurate clinical treatment.

Reported studies have indicated that the prognosis of MBC is better than that of IDC. Huober et al. found that the overall survival rates and 14-year distant recurrence-free intervals for invasive ductal tumours and medullary tumours of the full cohort were 57, 66, 64 and 76%, respectively (16). Similar findings were also found in the report of Dongjun Dai et al. (18). However, the results of the AMCB comparison between MBC and IDC were different. MBC occurs in patients of younger age; however, there was variation in the results of previous studies (5, 6, 19). Ethnic variations may play a role in such differences. Compared with patients who had an atypical characteristic, Rakha et al. (20) achieved better survival rates in patients with the typical characteristics of MBC. However, they found that the difference was not statistically significant. Conversely, Aksoy et al. (8) found that patients with atypical MBC presented significantly superior rates of recurrence and RFS. This may be associated with a smaller number of selected cases, and these patients have less tumour growth and angiogenesis with fewer lymphocytes and mononuclear infiltration. Some studies of immunohistochemical staining and gene expression analysis revealed that MBCs expressed a substantially higher fraction of triple-negative subtypes (ER, PR, and Her2) (21, 22). Considering local invasion, IDC has a more aggressive manner than MBC, and a previous study showed that MBC expressed a higher negative rate of lymph nodes than IDC (75.0% *vs* 47.9%, *P* = 0.0014) (23). In our study, the clinical features of 2,001 patients with MBC were analysed, and hormone receptor-negative cases comprised the majority. Her2 expression status was not included in this study as a prognostic factor. This was related to the number of cases that did not report Her2 expression before the 2010 SEER database. In our research, the prognosis of hormone receptor-negative AMCB patients was significantly worse than that of MBC patients. This was similar to the study of Shokouh et al. (24) and referred to the relationship of Ki67, Her2, p53, ER, and PR status and breast carcinoma subtypes. These previous studies have confirmed the possibility that hormone receptors will be used as prognostic indicators for AMCB.

The SEER database provides extensive data on breast cancer patients, which makes our study more convenient. However, there are limits to what we are able to do using this information. It is well known that there are no Her2 expression data in the SEER database prior to 2010. Thus, we are not able to group patients according to the 2015 St. Gallen consensus breast cancer classification; we can only separate the hormone receptor status into a single stratified study of the prognosis. In addition, different treatments for breast cancer have different effects on prognosis. The SEER database lacks data about patients who have received targeted therapy, endocrine therapy, and chemotherapy. Previous studies also used the SEER database and demonstrated that MBCs had distinctive clinicopathological characteristics, such as higher grade, larger tumour size, advanced stage, younger age at diagnosis, and a higher proportion of triple-negative breast cancer (7). The SEER database is more convenient for a specific pathologic review of specimens than for histological diagnosis.

In conclusion, the Cox analysis of multivariate and Kaplan–Meier analyses revealed that the prognostic factors of AMCB were worse than those of MBC in the hormone receptor-negative cohort. The prognostic factors were related to age, stage, and cancer-directed surgery. Finally, a novel nomogram based on the prognostic factors that combined all significant independent variables was established for the overall survival of AMCB patients and could be used to suggest a more applicable treatment.

## Data Availability Statement

Publicly available datasets were analyzed in this study. This data can be found here: https://seer.cancer.gov/data/.

## Ethics Statement

Our study was approved by the Ethics Committee of Changzheng Hospital. All patients provided written informed consent prior to enrolment in the study.

## Author Contributions

WQ, FQ, and MG participated in the conception and design of the study. FQ, LZW, and Y-SZ performed the statistical and bioinformatics analyses. WQ, FQ, MG, and Y-SZ coordinated, drafted, revised and finalized the manuscript. All authors contributed to the article and approved the submitted version.

## Conflict of Interest

The authors declare that the research was conducted in the absence of any commercial or financial relationships that could be construed as a potential conflict of interest.

## Abbreviations

SEER database, Surveillance, Epidemiology and End Results database; AJCC, American Joint Committee on Cancer; AMCB, atypical medullary carcinoma of the breast (MBC); MBC, medullary carcinoma of the breast; ER, estrogen receptor; PR, progesterone receptor; Her2, human epidermal growth factor receptor 2.
